# Evolution of large area TiS_2_-TiO_2_ heterostructures and S-doped TiO_2_ nano-sheets on titanium foils

**DOI:** 10.1038/s41598-019-53651-y

**Published:** 2019-11-29

**Authors:** S. Ahmad Etghani, E. Ansari, S. Mohajerzadeh

**Affiliations:** Thin Film and Nanoelectronic Lab, School of electrical and computer Eng., University of Tehran, Tehran, Iran

**Keywords:** Electrical and electronic engineering, Photocatalysis

## Abstract

We report a novel and facile method to synthesize sulfur-doped titanium oxide sheets and realize TiS_2_-TiO_2_ heterostructures by means of a sequential sulfurization and oxidation step in a dual-zone chemical vapor deposition furnace. The inclusion of chlorine and argon gases during the growth of such titanium-based compounds plays a critical role in the formation of desired geometries and crystalline structures. These heterostructures possess nano-whisker and nanosheet configurations, controlled by adjusting the growth parameters such as temperature, carrier gas and the sequencing between different steps of the growth. The evolution of these complex heterostructures has been investigated using Raman spectroscopy and EDS characterization. The presence of chlorine gas during the growth results in local TiS_2_ formation as well as faceted growth of TiO_2_ nanosheets through anatase to rutile phase change prohibition. The electron microscopy (TEM) images and diffraction pattern (SAED) characterization reveal the crystallinity and layered nature of grown structures, further demonstrating the 2D characteristics of S-doped nanosheets. The evolution of TiO_2_ on TiS_2_ heterostructures has also has been verified using XPS analysis. These highly featured nanostructures are suitable candidates to enhance the photocatalytic behavior of TiO_2_ nanostructures.

## Introduction

Transition Metal oxides (TMOs) with their versatility in crystallographic structures and solid-state properties are emerging materials for both research institutes and industrial enterprises. In the meantime, titanium dioxide possesses a large part of this research activity and investment enthusiasm. Although a great research stream is active on photocatalytic behaviors and optical applications of TiO_2_ polymorphs (Rutile, Anatase and Brookite)^1,2^, they have shown diverse physical and chemical properties, which make them a desirable candidate for applications such as food additives^3^, bioactive coatings^4^, drug delivery agents^5^ and also in flexible electronics^6^ in their nano-shaped forms.

The synthesis of TiO_2_ nanostructures in the forms of 1D features such as nanoribbons^7^, nanowires^8^ and nanotubes^9^ and 2D structures like nano-sheets^10^ as well as 0D nanoparticles^11^ has introduced new possibilities in their fields of applications. TiO_2_ nanostructures show enhancement in their photocatalytic activity (PCA) compared to their bulk counterparts^12^. Moreover, the higher surface to volume ratio of nanostructures brings a broader active field to absorb the incident ultra-violet (UV) spectra and their coupling with confined structures such as gold disks^13^. Along with the superior characteristics gained through size reduction, efforts have been made towards the growth of crystalline structures along with the desired facets due to the unique characteristics of special crystalline planes of TiO_2_^14^. In general, a faceted growth results from the minimization of surface energy which reveals surface-dependent features, depending on different Ti-O bonding and oxygen and titanium deficiency in the crystal structure. These dependencies to crystal surface play a dominant role in the anatase phase in which higher photocatalytic activities are expected. Furthermore, a better heterogeneous catalytic performance of {001} faceted grown sheets of the anatase phase of titanium-oxide towards {101} or {100} tailored structures is predicted owing to the presence of five coordinated Ti atoms, leading to higher surface energy^15,16^.

The tailored faceted synthesis of TiO_2_ has been investigated through both chemical and physical methods such as MOCVD^17^, CVD^18^, Molecular Beam Epitaxy^19^, solvothermal^20^ and hydrothermal^21^ methods while the two previous ones are the most favorable techniques. Using a capping agent such as Cl^− ^^22^, F^− ^^23^ and SO_4_^− ^^24^ and also flow of reactive gases such as chlorine can facilitate the evolution of facets with higher surface energies and higher indexes. The presence of reactive agents can affect the phase transformation of TiO_2_ polymorphs during the growth, as they can be classified into promoters and inhibitors of anatase to rutile phase changes^25^. The inclusion of reactive agents during the growth process can also act as a doping agent in the synthesized materials. The predetermined doping along with their post-growth residues can alter the solid-state properties of the grown structures. Owing to the critical role of TiO_2_ nanostructures in photocatalytic applications, the purpose of most investigations in this field is the enhancement of photoabsorption efficiency in the prepared samples and therefore, both metallic (i.e., Fe^3+^ and Cu^2+^)^26^ and nonmetallic (i.e., N, P, C and S) species^27,28^ are deployed. Ion implantation as a novel technique for doping of TiO_2_ nanostructures has been introduced by Li *et al*.^29^. Moreover, Song *et al*.^30^ have implemented the “Midas Touch” transformation through ion implantation of carbon and nitrogen dopants in the pristine TiO_2_ structures and enhanced the photoelectrical properties of the material.

Among the additives and dopants, sulfur as a non-metallic dopant has grasped the interest of researchers due to its contribution in adjusting TiO_2_ bandgap, as a result of sulfur substitution into oxygen sites of the lattice^31^. In order to achieve sulfurized TiO_2_ structures, different growth and doping techniques have been employed ranging from wet chemistry solvothermal^32^, hydrothermal^33^ and sol-gel synthesis^34^ to H_2_S treatment of TiO_2_ particles^35^. While CVD-based growth techniques have been widely employed to prepare S-doped samples^33^, the oxidative annealing of TiS_2_ films is known as a conventional method to achieve S-doped TiO_2_ nanostructures^28^. Jing *et al*.^36^ synthesized anatase TiO_2_ nanosheets through electrochemical exfoliation of TiS_2_ by lithium intercalation and subsequent sonication and centrifuging. The TiO_2_ nanosheets were then derived by a hydrothermal process in O_2_ purged solution.

In this work, 2D layered crystalline S-doped TiO_2_ nanosheets are grown on titanium substrates through a dual-zone CVD synthesis technique. A sequential procedure of the growth is implemented by sulfurization and subsequent oxidation in Ar and different Ar/Cl gas environments. The effect of chlorine gas on the growth mechanism has been investigated by changing the Ar:Cl gas ratios. The CVD furnace is equipped with a peripheral in-process load/unload apparatus for better monitoring and controlling the growth. The fabricated nanostructures have been thoroughly examined utilizing EDX, SEM, XPS, TEM, SAED, and Raman spectroscopy techniques. TiO_2_ surfaces covered with nanosheets were analyzed for the potential photocatalytic characteristics through temporal UV-Visible spectroscopic characterization of photodegradation in a Methylene Blue (MB) aqueous solution. The reaction kinetics of the photocatalytic process in nanostructured TiO_2_ samples have been studied using pseudo-first and second-order models.

## Materials and Methods

**Scheme 1:**
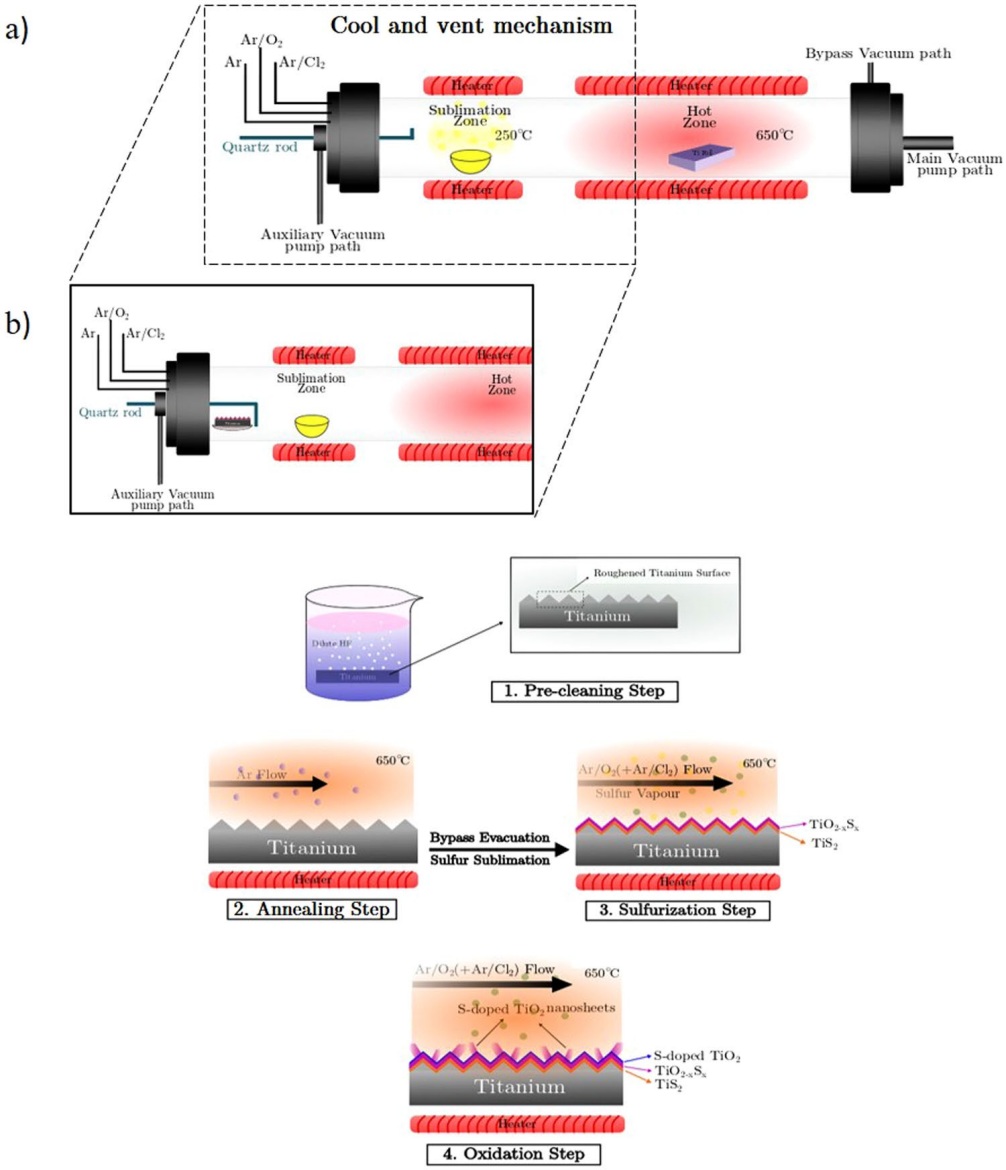
Schematics of growth apparatus and the proposed growth mechanism. (**a**) The enhanced CVD furnace for the growth of S-doped TiO_2_ nanostructures (dashed box depicts the cool and vent mechanism appended to this system for rapid sample pull-out). (**b**) The sequential growth process flow; surface roughening using HF solution, followed by annealing at 650 °C and sulfur exposure at the same temperature. The initial layer would be TiS_2_ whereas the top layer would turn into an oxidized layer during the oxidation step, forming heterostructures. S-doped TiO_2_ nanosheets are synthesized through the oxidation and subsequent cooling step.

The experiment is conducted in a dual-zone CVD tube furnace (depicted in Scheme 1a) in the presence of Ar/O_2_ gas flow. The furnace is evacuated with the aids of a mechanical rotary pump down to a base pressure of 20 mTorr for the initiation of the growth process. The sublimation zone of the quartz tube is located at one end of the tube to heat up the sulfur reservoir and to provide the necessary sulfur vapor precursor in the reactor. This zone is heated up to 250 °C with a slow rate of 20 °C/min and feeds the system with sulfur vapor, sublimated from 12 grams of S-powder. The hot zone maintains a higher temperature of 650 °C to host the growth and the TiO_2_ nanosheets are grown on a pre-cleaned titanium foil mounted on a quartz plate in this hot zone. Titanium foils (Good-Fellow, 99.6 + % purity) are used as the growth substrate and they are prepared through a preliminary cleaning step by immersing in a dilute HF (8:1 DI Water: HF) solution. Besides removing the native oxide layer formed on the Ti surface, a rather corrosive reaction between hydrofluoric acid (HF) and titanium increases the roughness of the foil surface to improve the growth and capturing probability of the inlet gases.

To achieve vertically-grown few-layered TiO_2_ nanosheets, the pre-cleaned Ti foils experience a sequential process (Scheme 1b). The synthesis begins with elevating the hot zone temperature to 650 °C in an Ar ambient with a gas flow of 15 sccm. After the initial pumping cycle, and to increase the pressure of the ambient gas and enrich the gas stream with sulfur, the pumping of the CVD reactor proceeds through the bypass evacuation mode which increases the furnace pressure up to 2 Torr (in the presence of Ar gas). This condition increases the presence of gaseous reactants on the surface of the Ti substrate. At this stage and by reaching the desired temperature in the hot zone, the pressure is maintained at the desired value and the sublimation of sulfur powder is started. This process is taken place in the flow of Ar/O_2_ gas with 15 scmm flow rates. The mixture of sulfur vapor and O_2_ gas plays a critical reactant role for the synthesis of TiS_2_ and TiS_2−x_ O_x_ on the surface of the Ti substrate. After the termination of the sulfurization process, the flow of Ar/O_2_ gas mixture is maintained. The growth of TiO_2_ nanosheets mainly proceeds at this oxidation step when the cooling of the sublimation zone progresses to reduce the presence of sulfur remnants on the surface of the sample. This condition is kept for one hour and accompanied by another cool-down step of the main hot zone of the furnace.

It has been shown^37^ that the presence of chlorine gas or chloride compounds during the growth process of titanium sulfide nanostructures can play an effective role because of the intermediary reaction between Ti and Cl species. Thereupon, the experiments are carried out by various Ar/Cl gas flows along with Ar/O_2_ mixture during the growth step. Two different mixtures of argon/chlorine gases (99.5/0.5 and 98/2) were used to trace the impact of the reactive agents in the nanostructure formation. To investigate the growth process in each step we have equipped the CVD apparatus with a loading rod which can transfer the sample from the hot zone to the cold zone in a smooth and rapid manner. Special care should be taken into account to ensure the vacuum is not affected during loading/unloading the sample during this transfer rod.

## Results and Discussion

**Figure 1:**
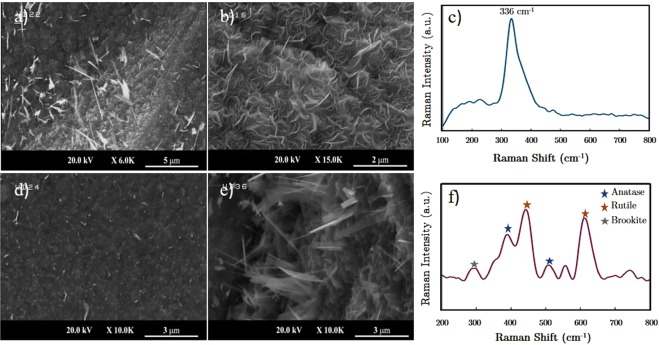
SEM images and corresponding Raman results of grown structures on pre-cleaned titanium substrates. (**a**) TiO_2_ nanowhiskers grown in Ar-Ar/O_2_ environment, (**b**) growth of TiS_2_ nanosheets in Ar/Cl_2_ (2%)-Ar/O_2_ environment, (**c**) Raman spectroscopy of (**b**), (**d**) Sample experiencing abrupt extraction by means of cool and vent apparatus after sulfurization, (**e**) The evolution of 3D sheets in the growth environment of Ar/Cl_2_ (0.5%)-Ar/O_2_ gas streams. (**f**) The observed Raman spectroscopy of (**e**), identifying the presence of Anatase, Rutile and Brookite phases of TiO_2_ structures.

Synthetization of S-doped TiO_2_ nanostructures was taken place in a sequential manner through sulfurization and oxidation steps which led to the formation of a nonstoichiometric titanium oxysulfide film as a groundwork for the growth of TiO_2_ sheets. The formation of free-standing nanostructures at the presence of three gases is considered as a backing substrate for the evolution of TiO_2_ sheets and whiskers. The SEM images of TiO_2_ nanosheets grown through the aforementioned procedure in the Ar/O_2_ gas flow are shown in Fig. 1a. TiO_2_ nano-whiskers have emerged from a bulky substrate that resembles condensing of 2D surfaces which form a thick uniform film. The thicker layers are detached on the upper surfaces and consequently, TiO_2_ nanostructures are flourished from these bulky and rough grains.

To enhance the sulfurization of titanium foils, the chlorine gas (Ar/Cl) is supplied into the growth reactor during this step as well as the oxidation one. The presence of chloride precursors leads to a significant improvement in the formation of titanium sulfide compounds. Introducing Ar/Cl (98:2) to the sulfurization step has led to a uniform growth of TiS_2_ nanosheets (Fig. 1b) and suppressed the formation of TiO_2_ films and nanostructures. The nanosheets synthesized in the chlorine-rich process have been studied using Raman spectroscopy and the corresponding data are depicted in Fig. 1c, where a sharp peak at 336 cm^−1^ corroborates the evolution of TiS_2_ nanosheets on the titanium surface.

The cease in the growth of titanium oxide and subsequent formation of TiS_2_ instead of TiO_2_ can be related to a probable reaction that is taking place between titanium foil and chlorine gas at an elevated temperature, which results in the regional formation of TiCl_4_ compounds on the Ti substrate. This hypothesis is based on the fact that metallic titanium shows a great tendency for the chlorination at temperatures about 300 °C which has been defined through thermodynamic states of reaction^38^. This reaction results in the gaseous TiCl_4_ byproduct through the reaction introduced in Eq. 1.

1 $$T{i}_{(s)}+2C{l}_{2(g)}=TiC{l}_{4(g)}$$

It is worth mentioning that the gaseous TiCl_4_ is widely used in producing titanium oxide^39^ and sulfide^40^ nanostructures. In the growth mechanism proposed in Scheme 1, the presence of sulfur vapor and oxygen gas in the sulfurization and further oxidation steps have made a competition role of TiCl_4_ as an intermediator between TiS_2_ and TiO_2_ nanostructure formation. It is well-known that the byproducts of the chlorine residues are easily removed from the surface of the sample and as a result, the presence of chloride compounds are not observed after the growth is complete^41^. The gaseous titanium tetrachloride is widely used as precursor in the growth of TiS_2_ nanostructures and its reaction with H_2_S gas in MOCVD furnaces has also been a favorite approach to arrive at different TiS_2_ films^42^. The massive TiS_2_ nanosheet formation in the chlorine-rich environment can be correlated to TiCl_4_ formation in the growth chamber and its further reaction with sulfur vapor generated through the sulfur sublimation. The presence of Ar/Cl mixture and dominancy of chloride compound formation suppresses the oxidation step and minimizes the undesired transition from TiS_2_ to TiO_2_ structures.

The amount of chlorine gas in the 2% Ar/Cl mixture is high enough to suppress the oxidation step and lead to desired TiS_2_ structures. In order to achieve TiO_2_ nanostructures, the chlorine gas ratio has been reduced to 0.5% to allow the reaction between titanium sulfide counterparts with oxygen, leading to TiO_2_ formation. The SEM image of titanium dioxide nanostructures formed in chlorine and oxygen environment is shown in Fig. 1e. The Raman spectra of this sample have been displayed in Fig. 1f, which depicts the presence of TiO_2_ phase in the nanostructured substrate and will be thoroughly studied in the following sections. The enhanced growth of nanostructures on the Ti substrate reveals the effectiveness of chlorine agent in the process. The role of chlorine gas in forming TiCl_4_ regions has improved the probability of local TiS_2_ formation by preparing a favorable growth substrate. The oxidation process coming along with this sulfide compound growth can lead to transitions to titanium oxide and to prepare an appropriate field of growth for TiO_2_ nanostructures and eventual TiO_2_/TiS_2_ heterostructures.

**Figure 2:**
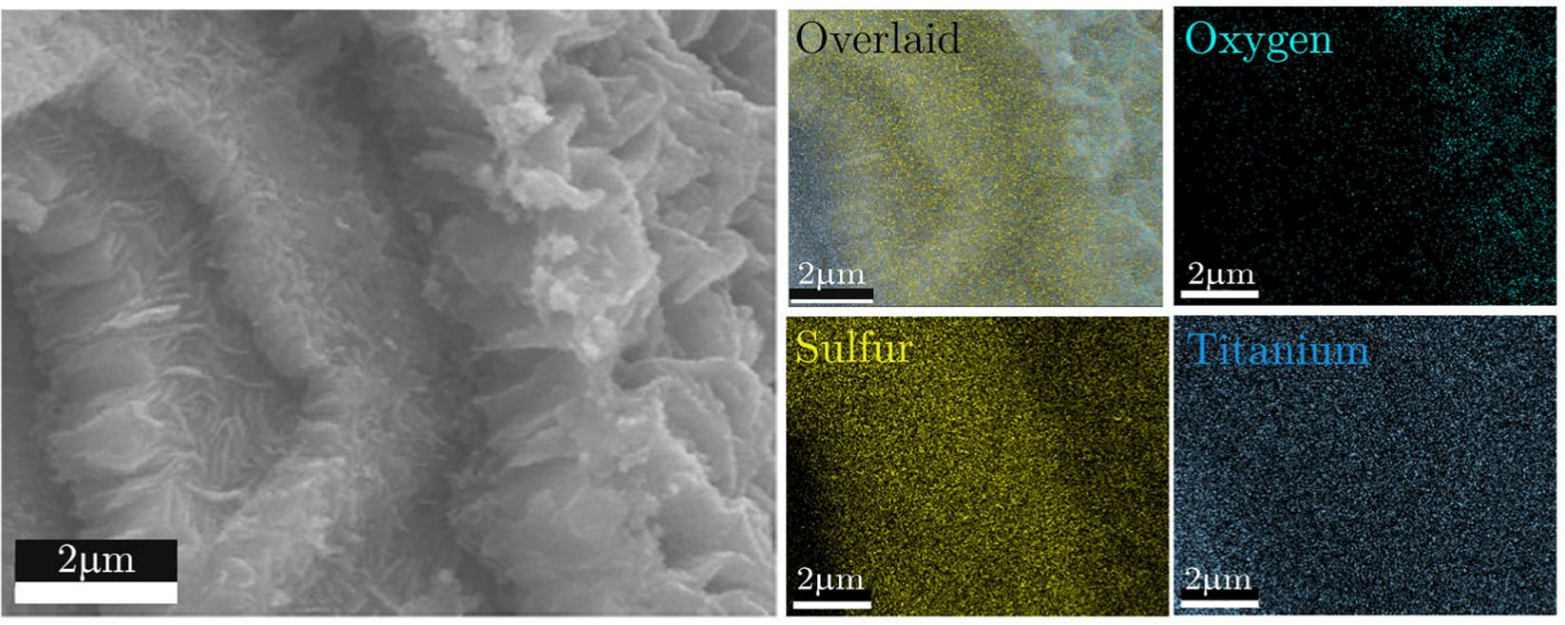
The elemental analysis (EDX) mapping of layered nanostructures grown on a pre-cleaned titanium substrate in Ar/Cl_2_ (0.5%). The highly layered structure is evident from the SEM image on the left side. While Ti is observed in all regions, the oxygen concentration is observed more in the upper layers, whereas sulfur shows a higher concentration at the lower layers.

To grasp a better insight about the grown structures, the upper surfaces of the samples are scratched off and the electron microscopy has been carried out on the peeled samples. The cross-sectional SEM image of the cleaved substrate revealed that the aforementioned sequential procedure has led to the growth of highly layered structures on the Ti substrate (Fig. 2) where highly dense sheets are created on top of each other. The EDX mapping reveals the nature of each layer and as seen in the sulfur elemental map of the sample where the lower layers are sulfur-rich, indicating the TiS_2_ sheets formation and the top features are more oxygen-rich, corresponding to TiO_2_ structures. The middle layer between the TiS_2_ nanosheets and the TiO_2_ layer synthesized during the oxidation layer has been formed due to possible strains during the growth of structures with different crystalline characteristics and is a sulfur-deficient TiS_2_ layer (TiS_2−x_ O_x_). This section has been synthesized in the sulfurization step in the presence of oxygen flow.

The presence of chlorine in the growth environment with a relative percentage of 2% seems to be critical in achieving such titanium-based heterostructures. The percentage of sulfur compound experience a noticeable diminution at upper layers. The innermost layer shows sulfur-rich layers of TiS_2_ sheets which were experienced a further EDX analysis presented in Fig. S1 by scotch tape transfer of grown layers on a silicon-based substrate. The topmost layer formed in the capacious nanosheets demonstrates a substituted behavior in the incorporation of oxygen atoms inside their structure. The excess of oxygen atoms can be traced by the EDX mapping of this element on the sample. The evolution of sulfur and oxygen atoms indicates that the upper layer has undergone an oxidation step where sulfide structures are transformed into titanium oxide form.

From the results of EDX analysis and Raman spectroscopy, it can be deduced that the TiO_2_ substrate on which TiO_2_ whiskers are grown has experienced a transition from TiS_2_ to TiO_2_ structures and a further phase transition from anatase to rutile, controlled mainly by the chlorine agent and the growth temperature. The transition of Titanium Sulfide to their titanium oxide counterpart and establishment of the growth in the oxidation step has provided an appropriate field of growth for S-doped TiO_2_ nanosheets. To evaluate this point, a manipulating apparatus is added to the CVD reactor which allows pulling out the samples from the hot zone after sulfurization step (depicted in Scheme 1a) in a modest time. A curved neck quartz rod is used to drag the sample’s boat towards the cold zone smoothly and after a period of cooling of 15 minutes. This cool and vent mechanism is an important step to avoid instantaneous exposure to air and oxygen. The SEM image of the samples presented in Fig. 1d shows that the growth of TiO_2_ whiskers was not established which suggests that the growth of nanostructures is performed mostly during the oxidation and cooling step.

The shifts in the Raman peaks depicted in Fig. 1f, sheds light on the presence of both two stable phases of TiO_2_ as anatase and rutile phases, on the cover layer of the synthesized structure. The anatase TiO_2_ structure has six vibrational modes as Γ = A_1g_ + 2B_1g_ + 3E_g_ signals are Raman active modes whereas four Raman active modes of Γ = A_1g_ + B_1g_ + B_2g_ + E_g_ are conceivable in the rutile phase. The Raman peaks of stable phases of TiO_2_ can be found in authentic papers^43,44^. The Raman spectra of the top layer of the sample shows five distinct peaks at 392, 447, 514, 561 and 614 cm^−1^ wavenumbers. The peaks located at 392 and 514 cm^−1^ wavenumbers can be interpreted as B_1g_ and B_1g_, A_1g_ modes of the Anatase phase. The dominant peaks of Raman analysis are devoted to 447 cm^−1^ and 614 cm^−1^ wavenumbers which are consistent with signals reported for rutile phase TiO_2_. The predominance of rutile TiO_2_ peaks has the relevance with the growth temperature of samples (650 °C) and the fact that TiO_2_ nanostructures experience a phase transformation from anatase to rutile configuration in the temperatures ranging from 600 °C to 1200 °C. A disjoint peak has appeared in the Raman spectra of the TiO_2_ samples at 561 cm^−1^ wavenumber which can be referred to as S_3_ molecules of sulfur, formed during the oxidation step or residues remained during the cooling process^45^. Also another peak in 288 cm^−1^ can be detected which can be attributed to the Brookite phase TiO_2_^46^.

**Figure 3:**
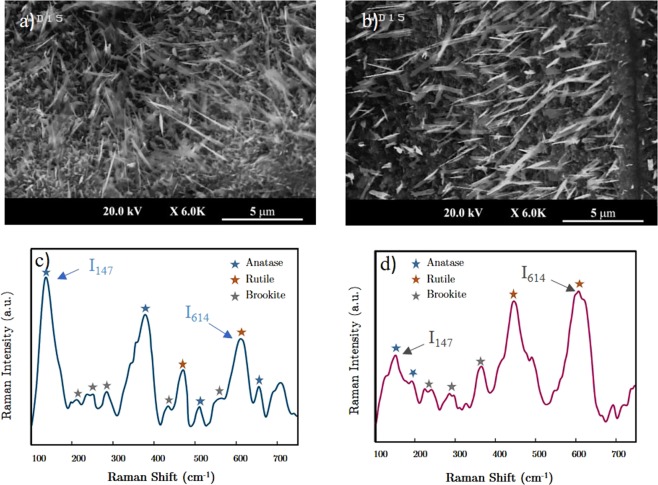
Effect of dominancy of each TiO_2_ stable phase in the amount of synthesized nanowhiskers coverage on the Titanium substrate. (**a**) SEM image of whiskers grown on higher anatase to rutile phase TiO_2_ films, (**b**) Raman spectroscopy of sample (**a**), (**c**) SEM image of whiskers grown on higher rutile to anatase phase TiO_2_ films, (**d**) Raman spectroscopy of sample (**c**). The ratio of Raman peaks at 614 cm^−1^ to 417 cm^−1^ wavenumbers can be considered as a measure to obtain a quantitative insight about this phase transition.

Although the anatase-rutile transition can be traced in the growth procedure, its effect on the formation of TiO_2_ nano-whiskers needs to be investigated. In order to understand this effect, two samples with different Anatase/rutile ratios were prepared by displacement of Ti substrate positions with respect to each other into the hot zone of the furnace. The resulted samples show slightly different surface morphology which led to variation in the number of whiskers grown on them as collected in Fig. 3. The nanostructures covering each sample propose a dissimilarity in the phase of the TiO_2_ substrate. The shifts in the Raman spectra correspond to various shifts which belong to highly complex structures. It is observed that the ratio of Raman peaks especially at 398 cm^−1^ and 147 cm^−1^ wavenumbers (anatase phase) to the peaks at 447 and 619 cm^−1^ wavenumbers (pertaining to rutile phase) is higher in the densified whisker grown sample which suggests that the anatase phase is a better field of growth for the vertically grown nanostructures.

In order to determine the anatase/rutile ratio in two samples, we have employed the formalism proposed by Clegg *et al*.^47^ in which the 147 cm^−1^ peak of Anatase phase and the 614 cm^−1^ peak of rutile phase were candidates for the representation of their content in the Raman spectroscopy result. As the appearance of the 147 cm^−1^ peak is very sensitive to the presence of anatase content, this methodology can be used for a better quantitative interpretation^48^. The anatase/rutile ratio is measured by means of dividing the intensity of Raman peaks at 147 cm^−1^ and 614 cm^−1^ and derived from the following equation;

2 $$R=\frac{Anatase\,content}{Rutile\,content}=\frac{{I}_{147}}{{I}_{614}}$$

The value of R, calculated for the sample with higher anatase content coverage (Fig. 3c) is 1.46 and this value decreases to 0.44 for the other sample which its Raman analysis is depicted in Fig. 3b. This observation leads us to conclude that the faceted TiO_2_ nanosheets are the consequence of anatase phase TiO_2_ formation on the layered film and anatase-rutile phase transformation is an unfavorable process which can terminate the growth procedure. This result highlights the role of chlorine gas in the growth process. The fact that the chlorine reactant is an inhibitor of anatase to rutile phase transition^49^ beside the aforementioned results proposes that the densified growth in the presence of Cl gas is a consequence of phase change prohibition enforced by the chlorine agent. This effect besides the intermediary role of chlorine in the formation of TiS_2_ sub-layers facilitates the growth of faceted structures.

**Figure 4:**
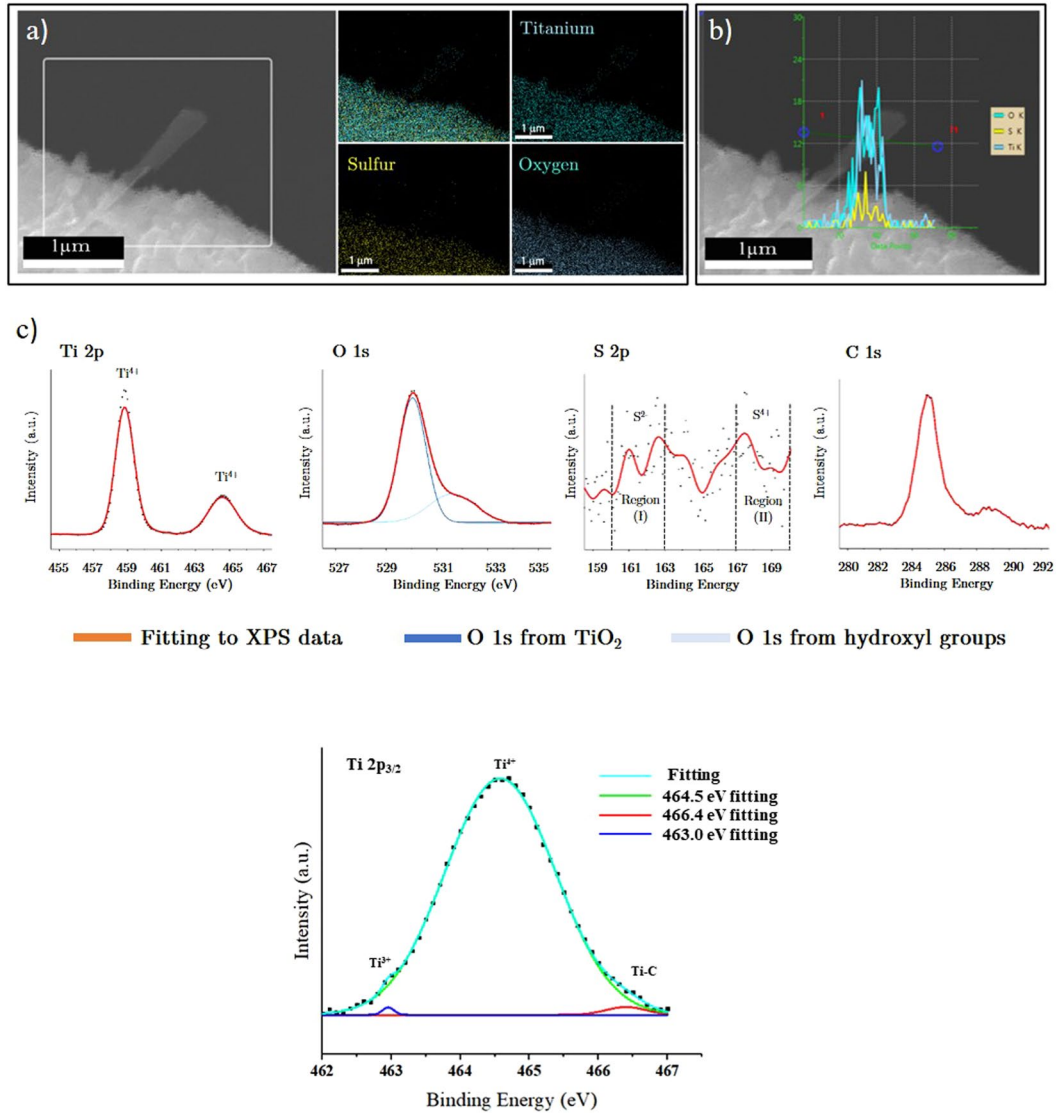
The EDX analysis and high-resolution XPS spectra measurement of S-doped TiO_2_ samples. (**a**) EDX mapping of titanium, sulfur and oxygen elements on nanowhiskers, (**b**) line scanning of single nanowhisker grown on TiO_2_ substrate. (**c**) The XPS analysis of nanowhiskers; the evolution of Ti-O bonds is observed from O1s peak. Since the XPS shows the very top surfaces of the sample, the presence of sulfur is less strong, although three regions of S bonds are observed in this figure. (**d**) The deconvoluted Ti 2p_3/2_ peaks which is assumed to be due to Ti^3+^ bonds.

The nano-whiskers were analyzed by energy dispersive spectroscopy (EDS) mapping to characterize the corresponding constituent elements. Figure 4a shows the presence of titanium, oxygen and also sulfur components in the sample. The mapping of each element elucidates the sulfur doping into titanium oxide nanostructures. A more exact examination of the sulfur portion in the structure can be achieved by line scanning of the whisker structures. The line-scan, provided in part (b), reveals the presence of sulfur on the scanned region and the O/Ti ratio below 2. The oxygen-deficient TiO_2_ nano-whiskers have been doped during the growth process by sulfur components which were presented either in the oxidation step of titanium sulfide substrate and its transition to titanium oxide or through their sulfur-rich seeds of growth.

Figure 4c shows high-resolution XPS results of the TiO_2_ sample. From the line-scanning EDX analysis presented in part (b), the contribution of sulfur species on the surface film formed on the fully processed samples after the oxidation step is minor and the XPS analysis can further confirm this conclusion. As depicted in S 2p XPS spectrum, although the measured peaks are weak, they are consistent with the reported peaks for sulfur ionic states in S-doped TiO_2_ and TiS_2_ films. By means of XPS measurements, the presence of S^2−^, S^4+^ and S^6+^ anionic and cationic species has been shown in the TiO_2_ samples prepared either by oxidation of TiS_2_ films^50^ or utilizing sulfur-based precursors during the growth process^51^. As stated by Han *et al*.^52^, sulfur S 2p peaks can be classified into two regions with binding energies of (I): 160–163 eV which present the S^2−^ anionic states contributing in the Ti-S bonds and replaced with the O^2−^ species, suggesting the substitution of oxygen atoms with sulfur^53^. The second region is region (II) which lays between 167 and 170 eV. This region is referred to as S^4+^ and S^6+^ cationic species replacing the Ti^4+^ states in the TiO_2_ crystal^54^. They can also be made from S–O and S=O bonds^55^ originated from the formation of SO_4_^2−^ or SO_3_^2−^ on the sample surface. The synthesized sample shows S 2p peaks in both regions. In the first region two peaks with binding energies of 162.6 and 160.9 eV were found which have good consistency with the S 2p_1/2_ and S 2p_3/2_ in the titanium and sulfur bonding in TiS_2_ compounds^56^ and express the replacement of S^2−^ anions in O^2−^ sites in the TiO_2_ lattice and the formation of slight doping in the sample. The other sulfur peak is found in the second region around 167.4 eV which represents the S^4+^ cationic species^57^. This evidence suggests that sulfur acts as both cationic and anionic doping in the synthesized sample through replacement with O^2−^ as well as Ti^4+^ atomic states. The XPS analysis does not show an indication of S^6+^ and SO^4−^ species.

The results obtained for titanium species indicate the significant peaks of Ti^4+^ in TiO_2_ with binding energies of 458.7 and 464.5 eV, which relates to Ti 2p_3/2_ and Ti 2p_1/2_ components^58^. Although the peaks are slightly shifted toward lower energies which can be related to the presence of sulfur species in the structure^51^, the predominance of TiO_2_ related Ti 2p peaks and the lack of TiS_2_ ones do not show strong sign of sulfur species. This can be related to the lean doping of sulfur atoms into the structure or the dominancy of S^4+^ in the doping profile.

Having a closer inspection of the Ti 2p_3/2_ peak, two marginal minor peaks can be extracted from the shoulder of the spectra. The extracted peaks have been depicted in Fig. 4d with binding energies of 463 eV and 466.4 eV. The sharp peak locating at 463 eV with FWHM of 0.17 belongs to the Ti^3+^ state of titanium. During the growth process leading to titanium oxide or sulfide, the presence of oxygen and sulfur in the gas state can dissociate the TiCl_4_ product to form byproducts such as TiCl_3_^59^ or TiOCl_2_^60^. The Ti^3+^ states participating in these compounds can be the origin of 463 eV peak. The other peak with a binding energy of 466.4 eV can be attributed to the presence of Ti-C bonding on the sample^61^.

The O 1 s spectra of the synthesized sample reconfirms the dominancy of TiO_2_ film formation with the 530 eV peak whose energy is in good agreement with the binding energy of titania^62^. As depicted in the XPS graph the spectrum is made with the combination of main TiO_2_ peak with the one with binding energy of 531.8 eV. This marginal peak can correspond to the oxygen atoms of hydroxyl groups or sulfate ones^63^, in which the hydroxyl formation on the substrate shows a better consistence with the results obtained from sulfur S 2p spectra. The C 1 s spectra is due to contamination and with the peak in 284.9 eV brings a 0.1 eV shift to reach the C-C bonding at 284.8 eV.

**Figure 5:**
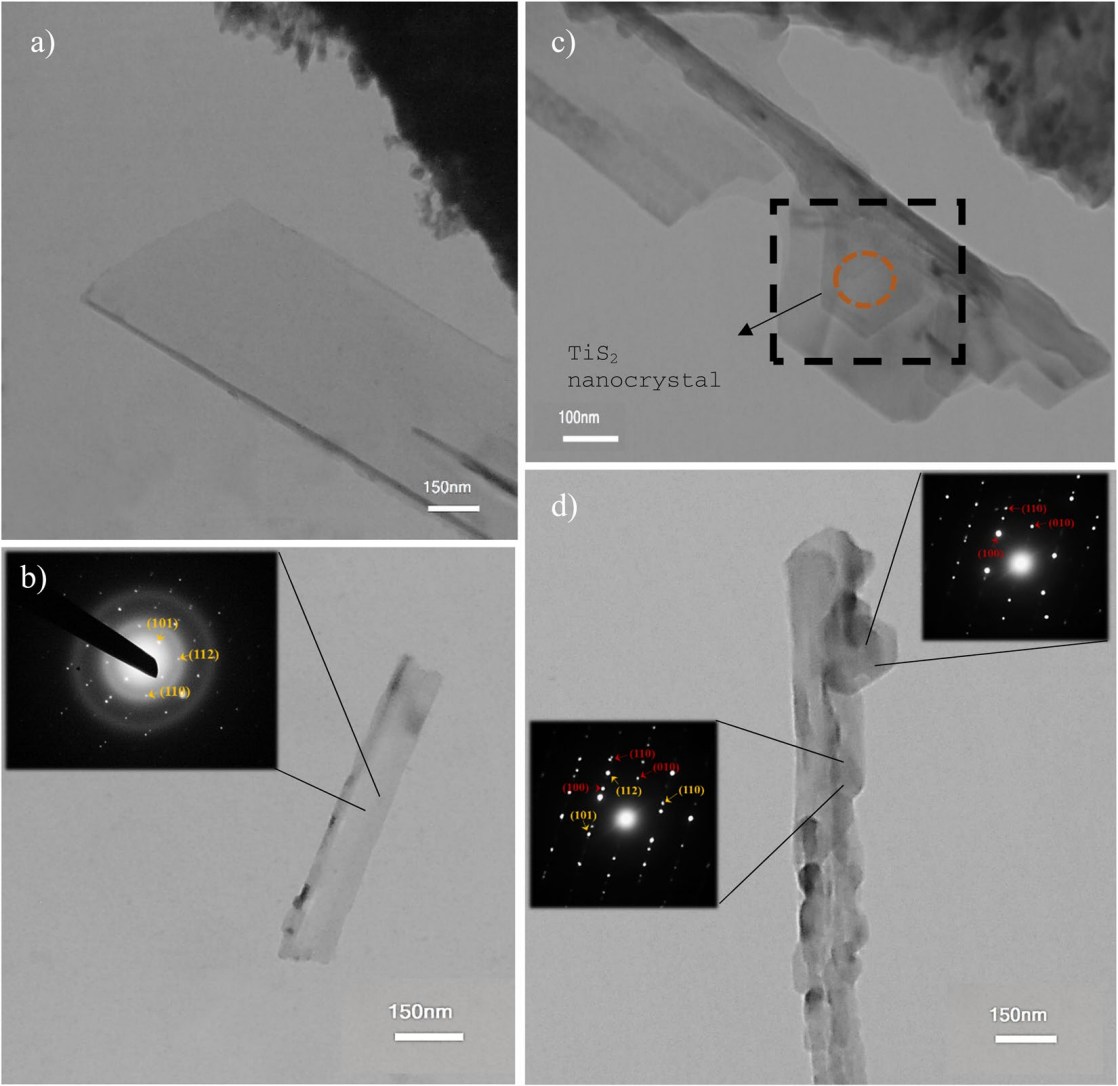
Collection of TEM images from various TiS_2_/TiO_2_ structures. (**a**) Ultrathin S-doped nanowhisker; (**b**) 2D layered nanowhisker, inset: SAED pattern and twinning in the diffraction pattern of nanowhiskers, (**c**) TiS_2_ nanocrystal emerged into the 1D structure, (**d**) nanowhiskers with their TiS_2_ seeds; insets depict the electron diffraction patterns of TiS_2_ nanocrystal and tailored nanowhisker. The green indexes belong to TiO_2_, while the red ones belong to TiO_2_.

The SEM images of synthesized structures show that the nanowhiskers are few-layered materials which are nearly transparent under electron beam exposure. A better inspection of these whiskers has been made using a Philips CM300 transmission electron microscopy, operating at 200 kV. Figure 5 depicts a bright-field TEM image of a whisker where a highly crystalline structure is evident. This image reveals the ultra-thin nature of nano-whiskers which are smoothly folded on their edges (Fig. 5a). The electron diffraction pattern (SAED) from the selected region displays the crystallinity of structures and whiskers. As the anatase TiO_2_, acting as the growth substrate for nano-whiskers, has a tetragonal unit-cell (P4_2_/mnm space group), its diffraction pattern can be formed in a parallel line configuration. The SAED pattern depicted in the inset of Fig. 5b appeared in a twining form which can be referred to overlaying parallel plane features and demonstrates the layered nature of nano-whiskers. The analyzed pattern exhibits excellent agreement with the anatase phase TiO_2_ nanostructures. The (101), (112) and (110) planes have been identified and indexed in the selected area diffraction patterns in this figure^64^.

The sequential growth and the gradual formation of TiO_2_ whiskers from TiS_2_ layers, studied by means of EDX analysis and Raman characterization. A clear delineation of this growth procedure can be traced in Fig. 5c in which the black dashed part of the TEM image depicts the TiS_2_ nanocrystal as the seed inside the nanowhisker. As highlighted with the dashed circle in this image, the hexagonal-shaped nanocrystal has tailored into 1D-shaped layer during the growth procedure which can shed light on the growth mechanism for TiO_2_ nanowhisker. The SAED patterns of these merged structures were characterized separately (Fig. 5d) in which the nanocrystal has a hexagonal diffraction pattern and the indexed pattern displays a good agreement to the one presented for TiS_2_ nanosheets^65^ the TiS_2_ nanocrystal is not isolated from the tailored nanowhisker and a heterostructure of stacked nanostructures can be assumed for the region being analyzed. Due to this fact, the neighbor structure has a trace in the pattern which is derived. This companionship can be followed in the SAED pattern of the tailored part of the material. This pattern displays concurrent presence of TiO_2_ and TiS_2_ planes which correspondingly indexed in the inset of Fig. 5d. This evidence reveals that the evolution of the TiS_2_ structures has resulted into the growth of these S-doped nanowhiskers and the desired heterostructure of TiS_2_ and TiO_2_ nanostructures.

The photocatalytic behavior of nanostructured samples has been investigated by inspecting the degradation of Methylene Blue (MB for short, purchased from Merck) aqueous solution under UV lamp illumination. The photo-catalysis tests were performed with 5 ppm MB solution with a pH level of 8.5, which due to the Point of Zero Charge (PZC) pH level of 6.8 for TiO_2_ nanoparticles^66^, an electrostatic absorption can be assumed between TiO_2_ negatively charged surface and MB dye^67^. The photocatalytic behavior of the TiO_2_ samples covered with nanowhiskers was compared with the TiO_2_ sample synthesized through 10 min oxidation step and extracted from the furnace by means of cool and vent apparatus depicted previously in Scheme 1 (part a). The reference sample has experienced an abrupt cool and vent mechanism after the primary 10 min oxidation which interrupts the process of nanostructure formation on the TiO_2_ surface. To evaluate the mass of covered surface on the samples, the surface of identical substrates peeled off with the aid of scotch tape which resulted in 4 micrograms coverage of synthesized material on the Titanium foil. The samples are constantly stirred in a 10 mL MB solution. All photodegradation tests have been performed under irradiation of 365 nm UV lamp with a power of 160 Watts and for a duration of 140 min. These tests have been performed after 40 minutes of dark adsorption equilibrium, the results of which have been provided in Fig. S2 of supplementary information. Also, the photodegradation of MB without catalysts has been conducted (Fig. S3), which shows a negligible removal of the dyestuff without catalytic reactions.

**Figure 6:**
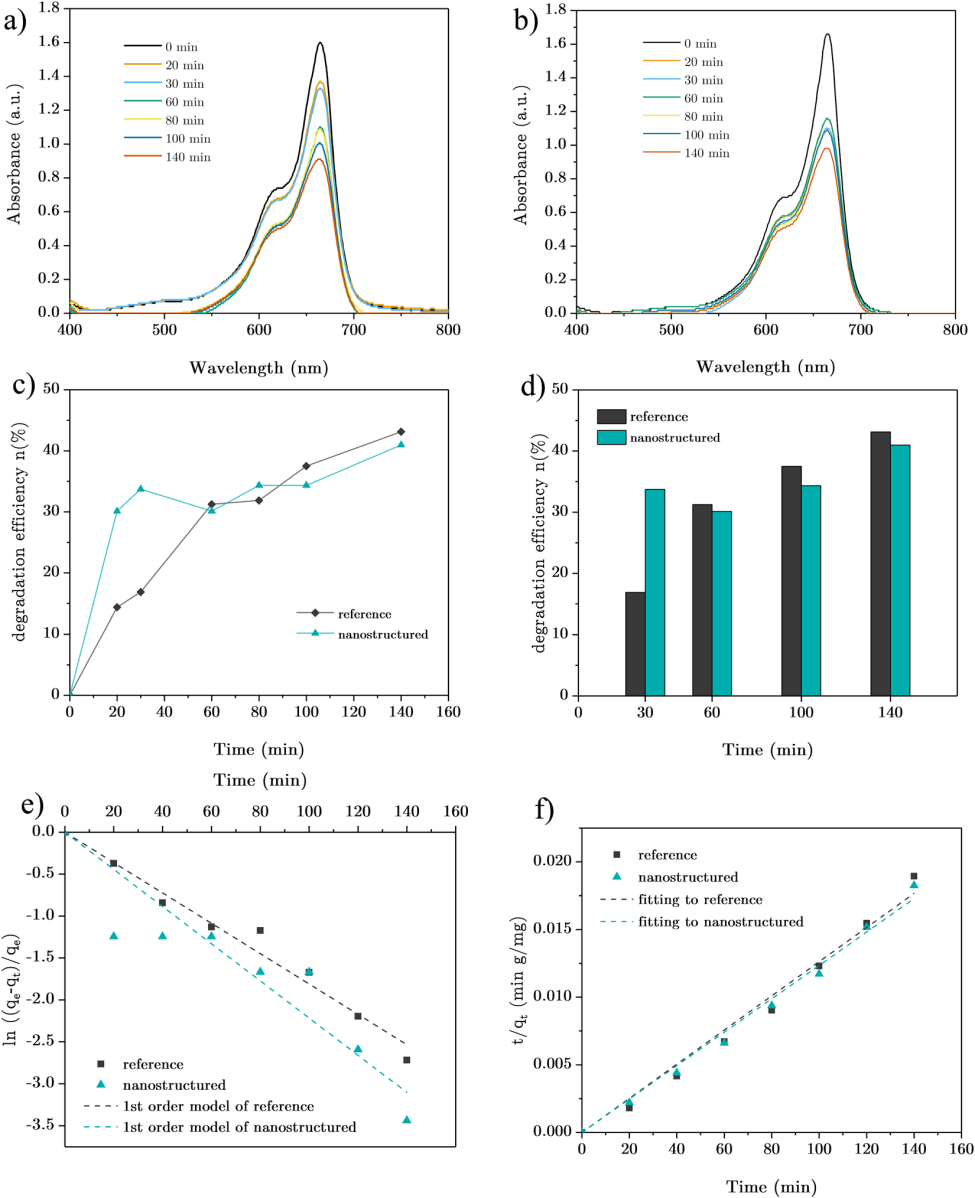
The photocatalytic behavior of the reference and nanostructured samples. (**a**) The absorption spectrum of 10ppm MB aqueous solution photo-degraded with reference sample TiO_2_ catalyst, (**b**) absorption spectra of solution in the presence of TiO_2_ nanowhisker sample, (**c**) temporal changes of photo-degradation efficiency during photocatalytic experiment, (**d**) bar chart of degradation efficiency variation in two photocatalytic processes, (**e**) adsorption kinetics of reference and nanostructured TiO_2_ samples modeled with pseudo first-order equation, (**f**) pseudo second-order fitting of the decontamination process.

The absorption spectrum of the degraded solution is presented in Fig. 6a,b for the reference and nanostructured samples, respectively. The UV-Visible characterization depicts the degradation of Methylene Blue dyes concentration through the photocatalytic action; although the complete refinement of aqueous solution shows a slow rate that reveals the fact of catalyst deficiency. A slight, blue-shifted displacement of the absorption peak in both samples was observed which can be referred to the Hypochromic effects occurring in the process of Methylene Blue N-demethylation^68^. The test conducted with higher concentration and volume of dyestuff and its subsequent solution (10 ppm and 20 mL) resulted in different shift values (4 nm for nanostructured sample and 1 nm in the case of reference substrate) in the course of 120 minute degradation time which lights up the interaction between nanowhiskers and contaminant dyes and the role of TiO_2_ nanostructures in cleansing MB solution and subsequent reduction and oxidation potential increment^69^.

The photocatalytic behavior of samples was evaluated through the calculation of the percentage of dye concentration removal using Eq. 3.

3 $$\eta ( \% )=\frac{{C}_{0}-{C}_{t}}{{C}_{0}}\times 100$$

where ŋ represents the degradation efficiency and C_0_ and C_t_ are initial concentration and concentration of MB dye at time t. The evolution of ŋ during the experiment time is plotted in Fig. 6c. Both samples show their highest photocatalytic activity in the first 30 mins interval and the rate of purification experiences diminution over time. Photocatalytic activity of pure and nanowhisker-covered samples presents the 34% degradation with the nanostructured surface against the 17% degradation in the presence of reference sample which yields in two-fold decontamination after 30 minutes. This result corroborates the efficiency of TiO_2_ nanowhiskers for MB dyes decomposition which can be related to their higher surface to volume ratio of photocatalytic sites. The other aspect of higher degradation rate in S-doped nanowhisker samples can be dealt with the role of sulfur doping. As the XPS analysis confirms, for the concurrent possible cationic and anionic nature of doping, the doping of sulfur atoms can bring oxygen and titanium vacancy states which results in electron-hole recombination suppression and further higher photocatalytic activity^70^.

The degradation rate of solutions experiences variations after the first 30 minutes of the experiment. Figure 6d plots temporal MB dye concentration variation (C_t_/C_0_) over the test period. As seen, the degradation mechanism differs in the decontamination process of two catalysts. The reference TiO_2_ exhibits linear characteristics over the experiment time which could be related to two possible mechanisms: (1) the diffusion-limiting regime governing the photocatalytic interaction between adsorbate and adsorbent and the apparent physisorption of dyes on the surface; (2) the adsorption of contaminants playing chemisorption properties but the deficiency of reactive sites limits the chemical adsorption process. Despite consistent slope found in the degradation rate of reference TiO_2_, the nanostructured surface exhibits a non-linear feature which specifies different catalytic kinetics governing their cleansing process. The temporal concentration changes depict that TiO_2_ nanostructures and MB dyes have interaction on the sample’s surface and contaminants have made chemisorption on TiO_2_ nanowhiskers. This adsorption has not resulted in the saturation of photocatalytic surface which could suppress the photo-degradation process^71^, but the lower rate of adsorption has affected the efficiency of nanostructured samples in degrading MB solution.

To better understand various mechanisms participating in the rate-controlling of photo-degradation process kinetic models can be employed. Due to different degradation characteristics derived from two samples, the heterogeneous photocatalytic processes have been modeled with both first-order and second-order kinetics. For this purpose, the Lagergren pseudo-first-order equation (Eq. 4)^72^ and also Ho’s pseudo-second-order formula (Eq. 5)^73^ are used for modeling the characteristics of both samples.

4 $$ln(\frac{{q}_{e}-\,{q}_{t}}{{q}_{e}})={k}_{1}t$$

5 $$\frac{t}{{q}_{t}}=\,\frac{1}{{k}_{2}{q}_{e}^{2}}+\frac{t}{{q}_{e}}$$

where q_e_ and q_t_ denote the adsorption capacity (mg/g) at equilibrium with respect to the time of the experiment (t). k_1_ (min^−1^) and k_2_ (g/mg. min^−1^) represent the rate constants of pseudo-first and second-order formalisms, respectively. Figure 6e depicts the pseudo-first-order fitting of the photo-degradation process of two catalysts by plotting temporal changes of ln((q_e_ − q_t_)/q_e_) versus time. The quality of fitting and the rate constant “k_1_” are extracted for each sample and presented in Table 1. The coefficient of determination gained from the nanostructured TiO_2_ plot, reveals the inability of first-order kinetics to fully interpret the governing mechanism for the photocatalytic degradation of MB in the presence of nanowhiskers. Nonetheless, the correlation of reference sample photo-degradation and pseudo-first-order model exhibits a good interpretation. Finally, part (f) represents the pseudo-second-order modeling of processes by plotting t/q_t_ over the experiment time period. The coefficient of determination for two fittings displays a distinct improvement in the case of nanostructured catalyst expressing that the MB dyes experience chemisorption on nanowhiskers, yielding in a descending scheme of photodegradation rate after 30 minutes of the process. On the other hand, the relative variation of determination of coefficient in the reference sample shows a minor enhancement. Through fitting of pseudo-first and second-order models to the reference sample, it is deduced that the adsorption of contaminants on the reference catalyst surface might have a slight chemical bonding, but it cannot make dominancy to limit the photocatalytic degradation process.

**Table 1:** Rate constants and the coefficient of determination (COD) calculated for two samples with the aid of pseudo-first and second-order models.

Rate Law	Photocatalytic sample	Rate Constant (K)	COD (R^2^)
Lagergren’s pseudo first order	Reference	0.0181 (min^−1^)	0.975
	Nanostructured	0.0222 (min^−1^)	0.835
Ho’s pseudo second order	Reference	1.262 × 10^−4^ (g min^−1^ mg^−1^)	0.984
	Nanostructured	1.235 × 10^−4^ (g min^−1^ mg^−1^)	0.991

**Figure 7:**
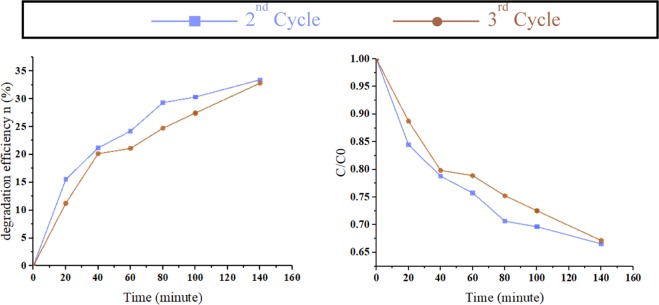
The second and third stages of cycling experiment for the degradation of MB in the presence of photocatalyst. In both cases, a monotonic rise in the degradation efficiency is observed, although the efficiency shows a slight reduction at the third cycle.

As stability is an important parameter in the photocatalyst efficiency, the photocatalyst has undergone a cycle test. The result of further cycles on the photocatalyst sample is presented in Fig. 7. These experiments were performed with the same recipe which has been conducted to the nanostructured sample (10 mL aqueous MB solution, 160 Watts, 365 nm UV lamp irradiation in 140 min duration).

The cycle experiment reveals that the photodegradation of MB in the presence of photocatalyst decreased from about 40 percent degradation after 140 min in the first cycle presented in Fig. 6c to 33.5% in the second cycle and 33% in the third one. On the other hand, this trend of decrements exhibits a saturation in the possible efficiency gaining from the presented photocatalyst sample.

## Summary and Conclusion

Single-crystalline sulfur-doped TiO_2_ nano-whiskers were grown in a dual-zone CVD furnace. The growth was monitored by means of loading/unloading mechanism being installed on the CVD reactor. The synthesis of TiO_2_ nano-whiskers was held in a sulfur-rich environment, leading to sulfur doping of nanostructures. The elemental analysis by EDX along with Raman spectroscopy revealed that the growth substrate for nano-whiskers formed in a layered stack of TiS_2_ and TiO_2_ films. This layered structure is a result of the sequential growth method, where the surface-featured titanium substrate experienced a sulfurization and subsequent oxidation step. Our investigations show that nanowhiskers are formed TiS_2_ seeds through oxidation and cooling steps.

The observed enhancements in the growth of nanostructures are believed to be due to the introduction of chlorine agents. The reaction between Ti substrate and chlorine gas provides the appropriate medium for the local growth of TiS_2_ sheets leading to confined TiO_2−x_ S_x_ sheets. On the other hand, the SEM and Raman spectroscopy findings state that the faceted growth of TiO_2_ nanowhiskers can be retained from the formation of anatase TiO_2_ layers and the chlorine gas can act as a prohibitory agent in the anatase to rutile phase transformation. Consequently, the chlorine agent plays an effective role in the growth of TiO_2_ nanostructures. The evolution of TiS_2_ layers and their transformation into TiO_2_ films and further nanosheets growth would eventually lead to S-doped heterostructures. The EDX mapping and line scanning of TiO_2_ nano-whiskers showed the presence of Sulfur doping. Also, transmission electron microscopy shows the ultrathin nature of synthesized nanosheets and the fact that the TiO_2_ whiskers are formed in a layered nature, proposing that such nano-whiskers are 2D sheets made of sulfur-doped titanium dioxide.

The photocatalytic behavior of TiO_2_ substrates covered with sulfur-doped nanosheets was studied by the so-called aqueous Methylene blue (MB) decontamination photocatalytic process. These nanostructured samples showed a two-fold enhancement in the photocatalytic activity in comparison with the reference TiO_2_ substrate during the first interval of 30 mins. The interpretation of degradation of MB dyes in the presence of nanostructures had been achieved through second-order fitting and pseudo-second-order kinetic modeling. These results express possible chemisorption of dyestuff on the TiO_2_ nanowhiskers during the photocatalytic procedure. We believe much higher photocatalytic degradation efficiency can be achieved by increasing the TiO_2_ nanosheet surface participating in the photocatalytic procedure.

## Supplementary information

Supplementary Information
