# Supplementary material for: Evolution of large area TiS2-TiO2 heterostructures and S-doped TiO2 nano-sheets on titanium foils
Source: Sci Rep. 2019 Nov 29;9:17943. doi: 10.1038/s41598-019-53651-y (PMC6884512; doi:10.1038/s41598-019-53651-y)
Supplement: Supplementary file 1 — Supplementary Information [file 41598_2019_53651_MOESM1_ESM.docx]

**Evolution of large area TiS_2_-TiO_2_ heterostructures and S-doped TiO_2_ nano-sheets on titanium foils**

**Supplementary Information**

**1-The EDX analysis of nanosheets covering innermost layer of nanostructured samples**

The peeling of the layers has been achieved using conventional Scotch tape technique where the tape is stuck to the structure and the sheets are separated from the main body. To prepare samples for electron microscopy analysis, the adhesive tape is contacted to the sample and the innermost layer is transferred to a Si substrate coated with 5 nanometers of gold. The transferred sheets shown in Fig.S1 have hexagonal shape which is known as the crystal structure of TiS_2_ sheets and the EDX mapping reveals the elemental percentage of the sheets. As depicted in the map, the elemental percentage of S element is almost double the Ti atoms, corroborating the formation of TiS_2_ sheets on the innermost layers of grown films. The trace of other elements found on the map can be interpreted as the residues remained or buried within the sample during transferring of sheets.


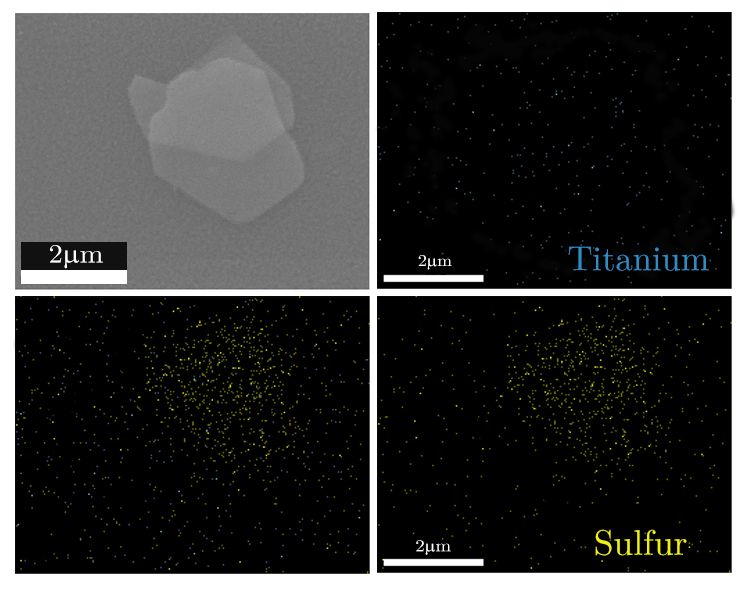


**Figure S1:** The SEM image of hexagonal sheets of TiS_2_ nanostructures followed by their EDX mapping for the samples prepared in Ar/Cl_2_ (0.5%) environment.

**2- Dark adsorption Equilibrium Experiment**

The dark adsorption equilibrium experiment had been performed for the samples under test. The samples were undergone 40 minutes of dark confrontation with the MB solution and the data was presented after that the illumination of light source took place. The 40 minutes of dark experience realized from the dark equilibrium experiment for a sample and its data is presented in Fig. S2. as depicted in these plots, the sample reaches to its equilibrium after 40 minutes in its first cycle of photodegradation. This evidence results that the photocatalytic samples reach the adsorption equilibrium in 40 minutes duration.


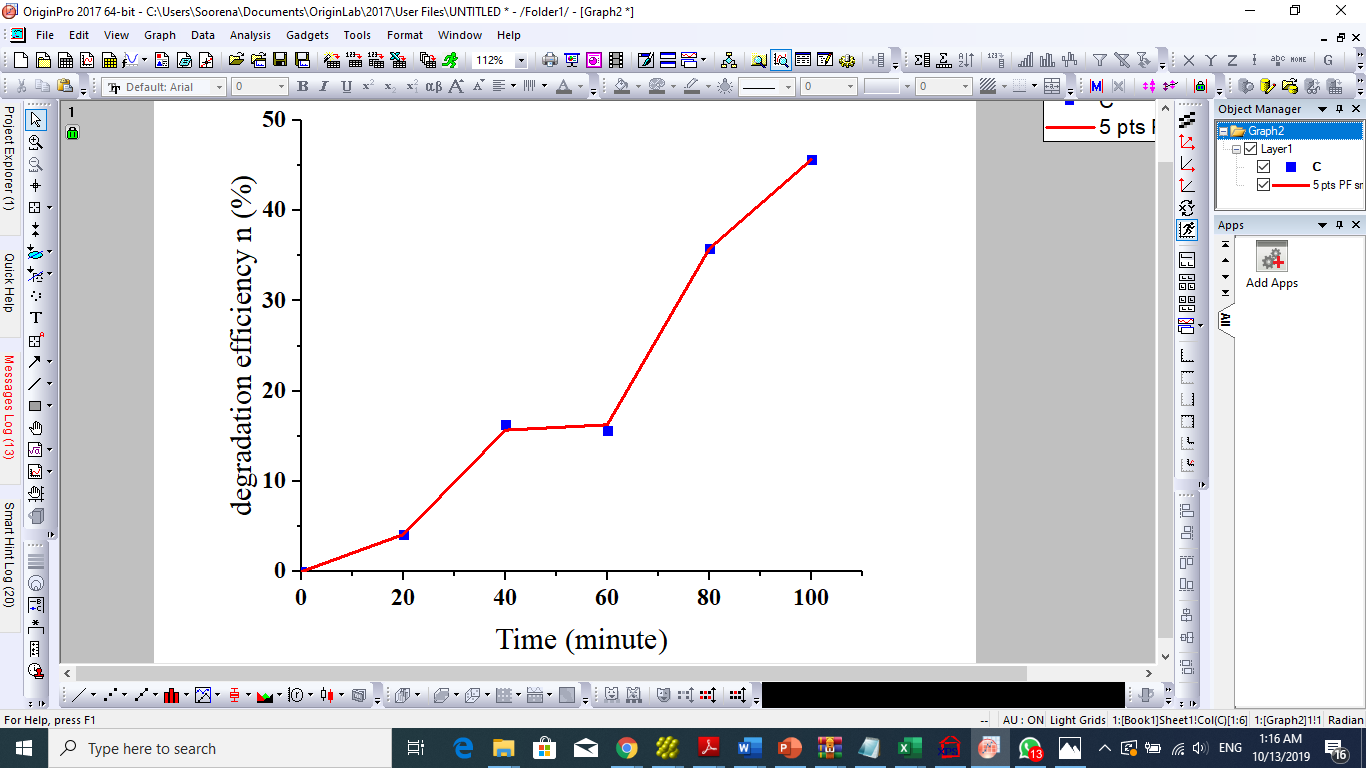

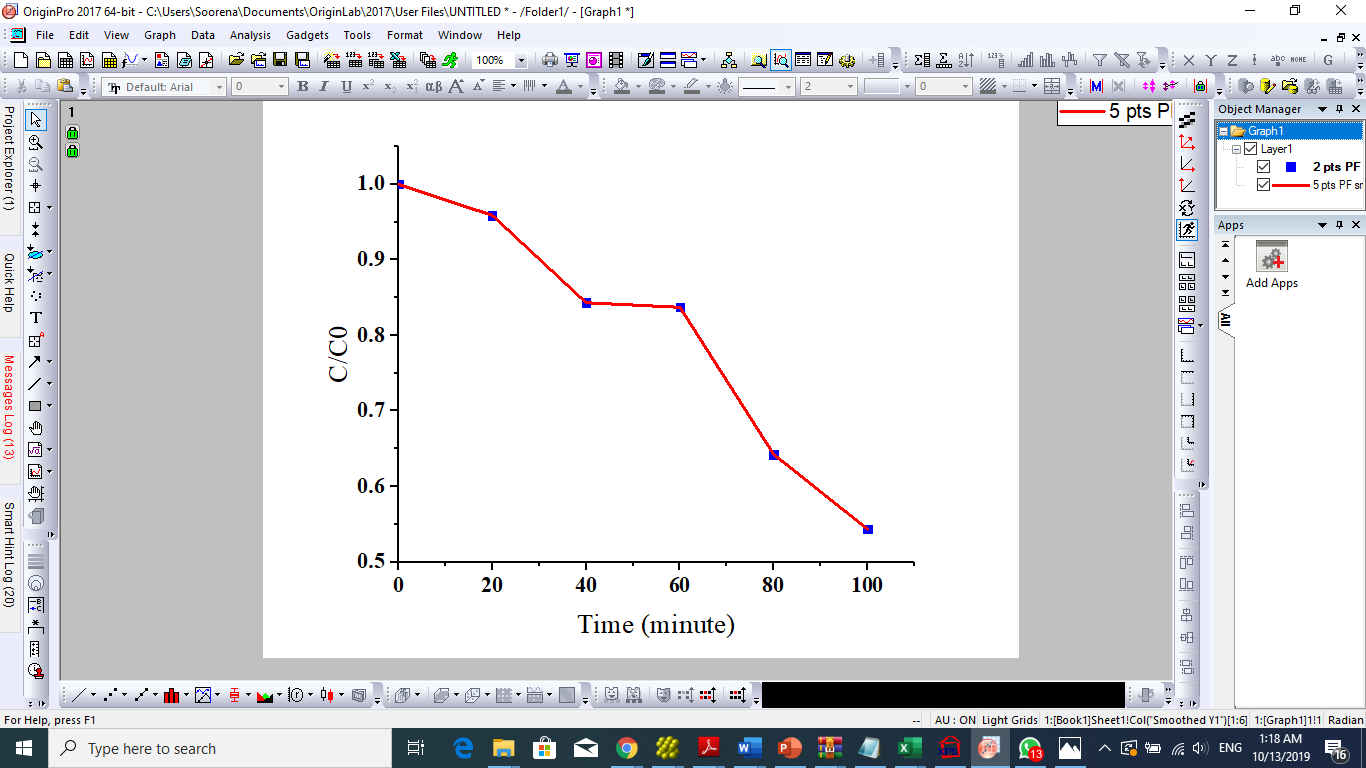


dark

Illumination

dark

Illumination

**Figure S2:** The dark equilibrium experiment results for the photocatalyst sample.

**3- Control experiment for the MB photodegradation without catalyst**

In order to measure the function of dyestuff without the presence of photocatalyst and grasping background data of the photocatalytic tests, 10 mL of MB solution (without catalyst) were investigated through the photocatalytic routine (160 Watts, 365 nm UV lamp irradiation in 140 minutes duration). The temporal photodegradation plot of this experiment is presented in Fig. S3. This measurement shows that the degradation of dyestuff is about 1 percent (C/C_0_ =0.989) in 140 minutes of UV irradiation. This degradation is negligible due to variations can be found in any experiment.


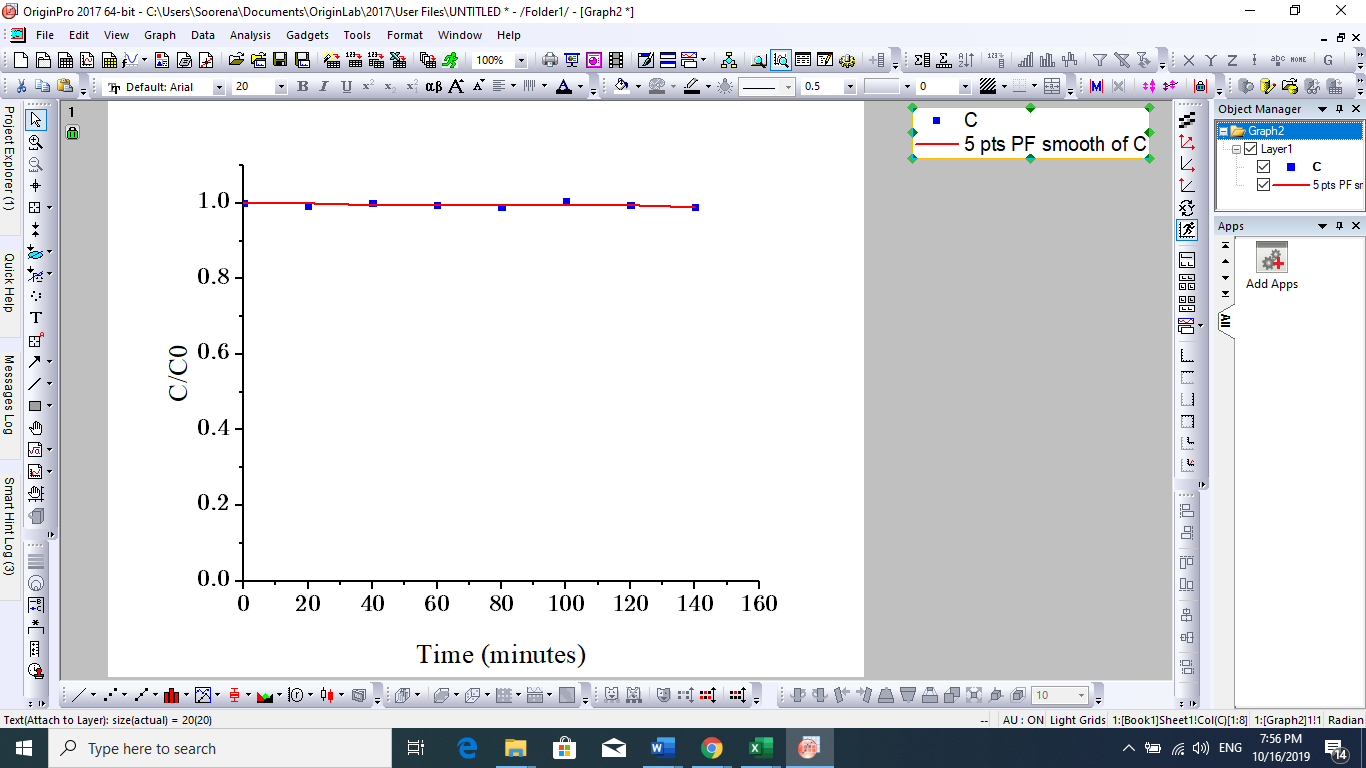


**Figure S3**: the temporal plot of MB degradation without the presence of photocatalyst
